# The epidemiology and clinical features of rickettsial diseases in North Queensland, Australia: Implications for patient identification and management

**DOI:** 10.1371/journal.pntd.0007583

**Published:** 2019-07-18

**Authors:** Alexandra G. A. Stewart, Simon Smith, Enzo Binotto, William J. H. McBride, Josh Hanson

**Affiliations:** 1 Department of Medicine, Cairns Hospital, Cairns, Australia; 2 College of Medicine and Dentistry, James Cook University, Cairns, Australia; 3 Kirby Institute, University of New South Wales, Sydney, Australia; Baylor College of Medicine, UNITED STATES

## Abstract

**Background:**

Rickettsial infections are a common cause of hospitalization in tropical settings, although early diagnosis is challenging in the rural locations where these infections are usually seen.

**Methods:**

This retrospective, clinical audit of microbiologically-confirmed cases of scrub typhus or spotted fever group (SFG) rickettsial infection between 1997 and 2016 was performed a tertiary referral hospital in tropical Australia. Clinical, laboratory and radiological findings at presentation were correlated with the patients’ subsequent clinical course.

**Results:**

There were 135 locally-acquired cases (95 scrub typhus, 37 SFG, 3 undifferentiated). There were nine hospitalizations during the first 5 years of the study period and 81 in the last 5 years (p for trend = 0.003). Eighteen (13%) of the 135 cases required ICU admission, all of whom were adults. A greater proportion of patients with SFG infection required ICU support (8/37 (22%) compared with 10/95 (11%) scrub typhus cases), although this difference did not reach statistical significance (p = 0.10). Three (8%) of the 37 patients with SFG infection had severe disease (1 died, 2 developed permanent disability) versus 0/95 scrub typhus patients (p = 0.02). Adults with a high admission qSOFA score (≥2) had an odds ratio (OR) of 19 (95% CI:4.8–74.5) for subsequent ICU admission (p<0.001); adults with a high NEWS2 score (≥7) had an OR of 14.3 (95% CI:4.5–45.32) for ICU admission (p<0.001). A patient’s respiratory rate at presentation had strong prognostic utility: if an adult had an admission respiratory rate <22 breaths/minute, the negative predictive value for subsequent ICU admission was 95% (95% CI 88–99).

**Conclusions:**

In the well-resourced Australian health system outcomes are excellent, but the local burden of rickettsial disease appears to be increasing and the clinical phenotype of SFG infections may be more severe than previously believed. Simple, clinical assessment on admission has prognostic utility and may be used to guide management.

## Author summary

Rickettsial infections are a common cause of hospitalization in tropical settings, although early, definitive diagnosis is challenging in the rural and remote locations where they are usually seen. It is important to recognise rickettsial infections early in their disease course as they can lead to life-threatening multi-organ failure if specific anti-rickettsial antimicrobial therapy is not prescribed promptly. In tropical Australia, scrub typhus and spotted fever group (SFG) rickettsiae are the dominant rickettsial pathogens and this twenty-year retrospective series examines the clinical and laboratory findings which might facilitate their recognition. The study highlights the infections’ increasing local clinical burden and reports that over 20% of the SFG cases in the series required Intensive Care Unit (ICU) admission, suggesting that severe SFG disease may be more common than previously believed. Simple, clinical prediction scores—calculated at presentation—identified patients who would subsequently require ICU admission. Importantly, they were also able to identify patients at low risk of disease progression. These entirely clinical scores—which can be calculated rapidly at the bedside—have the potential to facilitate the management of patients with scrub typhus and SFG infection, particularly in resource-limited settings which have the greatest burden of disease.

## Introduction

Rickettsial infections are a common cause of hospitalization in tropical settings [1–4], although early, definitive diagnosis is challenging in the rural locations where these infections are usually seen [5]. Antibiotic therapy is highly effective if started early in the disease course [6, 7], although anti-rickettsial agents have limited activity against other serious pathogens–including malaria and bacterial sepsis–that can have similar presentations [2, 8].

The clinical manifestations of rickettsial disease range from a mild, self-limiting illness to life-threatening multi-organ failure, although there are surprisingly few series that report the diseases’ clinical findings in a detailed manner [8–11]. Identifying the features of a patient’s presentation that increase the likelihood of rickettsial infection would help clinicians decide when to add anti-rickettsial therapy to empirical regimens. Meanwhile, identifying the features associated with the development of life-threatening infection would help expedite transfer of the high-risk patient to referral centers where more advanced supportive care is available [11].

In Far North Queensland, in tropical Australia, acute undifferentiated fever is a common clinical presentation [12]. Scrub typhus and spotted fever group (SFG) rickettsial infections, namely *Rickettsia australis* (Queensland tick typhus) and *Rickettsia honei* should be considered in the differential diagnosis, although other locally endemic infections–including leptospirosis, melioidosis and Q fever–can have a very similar presentation [13–16]. Rickettsial diseases were common in the region in the mid-twentieth century [17, 18], but more recently only small case series have been published [10, 19, 20]. This might suggest that their incidence has declined in the region, but detailed study of the infections’ temporospatial epidemiology has been lacking.

This twenty-year retrospective review was therefore performed to examine the issue more systematically. The study also examined the clinical and laboratory features of these infections that might be used to facilitate their diagnosis and to expedite the identification of the patients at risk of life-threatening disease.

## Methods

This retrospective, clinical audit was performed at Cairns Hospital, a 531-bed tertiary referral hospital for the Far North Queensland region, an area of 204,255 km^2^ with a population of 279,354 [21]. Patients were eligible for inclusion in the study if they were admitted to the hospital between January 1, 1997 and December 31, 2016 with a clinical diagnosis of scrub typhus or SFG infection accompanied by a positive polymerase chain reaction (PCR), a four-fold rise in titres of paired serological samples (confirmed infection) or a single serological titre ≥128 with a clinically compatible syndrome (two or more of fever, rash, eschar, myalgia or headache with no convincing alternative diagnosis) (probable infection). Patients were excluded if a non-rickettsial diagnosis was felt more likely or if the illness was considered to have been contracted outside the region.

The patients' medical records were reviewed to collect their demographic data, risk factors, clinical presentation, comorbidities, management and disease course. The antibiotic therapy was compared to national recommendations (seven days of doxycycline 100mg twice daily or azithromycin 500mg on day one, then 250mg daily for a further four days) [22]. The statewide laboratory information system was accessed to collect the results of the haematology, biochemistry and microbiology investigations. Hospital records were used to review radiology and echocardiography results.

Severe disease was defined as the requirement for intensive care unit (ICU) admission. Clinical prediction scores (qSOFA and NEWS2) at presentation were determined retrospectively in adult patients (≥16 years of age) and correlated with their subsequent clinical course. The cut-off for a high qSOFA score was ≥2 and a high NEWS2 score was ≥7 [23, 24].

The results of all rickettsial diagnostic tests requested in the public health system of Far North Queensland were also examined. A variety of serological techniques were used during the study period (S1 Table). From 1997 to mid-1999, the state’s reference laboratory (Forensic and Scientific Services, Brisbane, Australia) manufactured in-house slides for indirect immunofluorescence assay (IFA). From mid-1999 to mid-2009 an enzyme immunoassay (EIA) (PanBio, Brisbane, Australia) was used as a screening test, with positive EIA samples sent to the reference laboratory for confirmatory IFA. From June 2009 to December 2016 commercial IFA slides (BioCell Diagnostics, Baltimore, USA) were used. PCR targeting the 17 kDa antigen gene for SFG rickettsiae and the 56 kDa scrub typhus antigen gene for *Orientia tsutsugamushi* were available from 1998 [25, 26]. Although murine typhus has been reported in the region in the past [27], from 1999 typhus group IgG total antibody testing was not available, precluding definitive differentiation of SFG infections from murine typhus. However, 21/26 serological tests for murine typhus performed in the region between 1998 and 1999 were negative; while the 5 reactive tests all had a titre of <128, and 4 were also reactive for either SFG or scrub typhus. Disease incidence between 1998 (the first full calendar year for which data were available) and 2016 was determined using Australian Bureau of Statistics data [21].

### Statistical analysis

Data were de-identified, entered into an electronic database (Microsoft Excel 2016, Microsoft, Redmond, WA, USA) and analysed using statistical software (Stata version 14.0, StataCorp LLC, College Station, TX, USA). Univariate analysis was performed using the Kruskal-Wallis and chi-squared tests. Continuous variables with an area under the receiver operating characteristic (AUROC) curve of > 0.7 in univariate analysis were selected for multivariate analysis. These continuous variables were transformed into binary variables—using cut-offs based on common clinical usage—with multivariate analysis performed using backwards stepwise logistic regression.

### Ethics statement

The Far North Queensland Human Research Ethics Committee provided ethical approval for the study (HREC/17/QCH/66–1148 QA). As the data were retrospective and de-identified, the Committee waived the requirement for informed consent.

## Results

**Fig 1:**
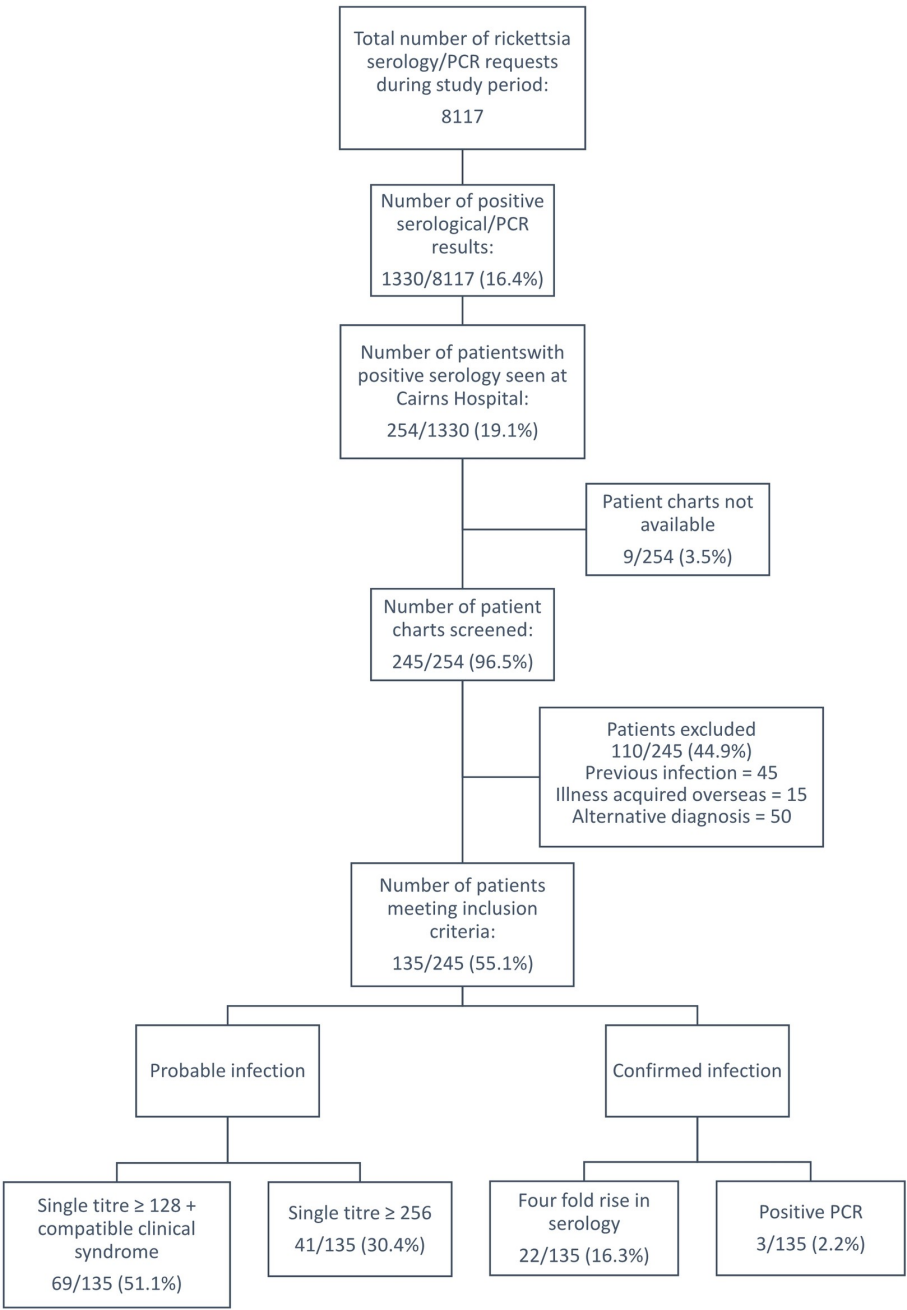
Data collection.

There were 254 patients who satisfied the inclusion criteria, 9 medical records were unavailable, leaving 245 patients for review. Of these 245, 135 met the pre-specified criteria for a locally acquired rickettsial infection (Fig 1). Scrub typhus (95 (70%) cases) was more common than SFG infection (37 (27%) cases); in three (2%) cases with clinical features of rickettsial infection, the titres for *Orientia tsutsugamushi* and SFG infection were both 128, precluding definitive identification of the causative organism.

There were 25 (19%) confirmed cases (positive PCR or four-fold rise in paired sera) in the series. Patients admitted to ICU were more likely to be confirmed cases (9/18 (50%) versus 16/117 (14%) non-ICU cases, p < 0.001), likely reflecting the greater interest of clinicians in determining the aetiology of a more serious illness. Among patients with scrub typhus, 14/95 (15%) had confirmed disease compared with 11/37 (30%) SFG cases (p = 0.08). Meanwhile, 4/11 (44%) SFG cases admitted to ICU had confirmed disease, compared with 5/14 (36%) scrub typhus patients (p = 1.00).

### Temporal epidemiology

The number of patients admitted to Cairns Hospital with rickettsial infections increased during the study period (all rickettsial infections (p for trend = 0.003), scrub typhus (p for trend = 0.001) and SFG infection (p for trend = 0.04)). There were nine hospitalizations during the first five years of the study period and 81 in the last five years. There was no observed seasonal trend in patient presentation: 69/135 (51%) presented during the 6-month November-April wet season while 66/135 (49%) presented during the May-October dry season.

**Fig 2:**
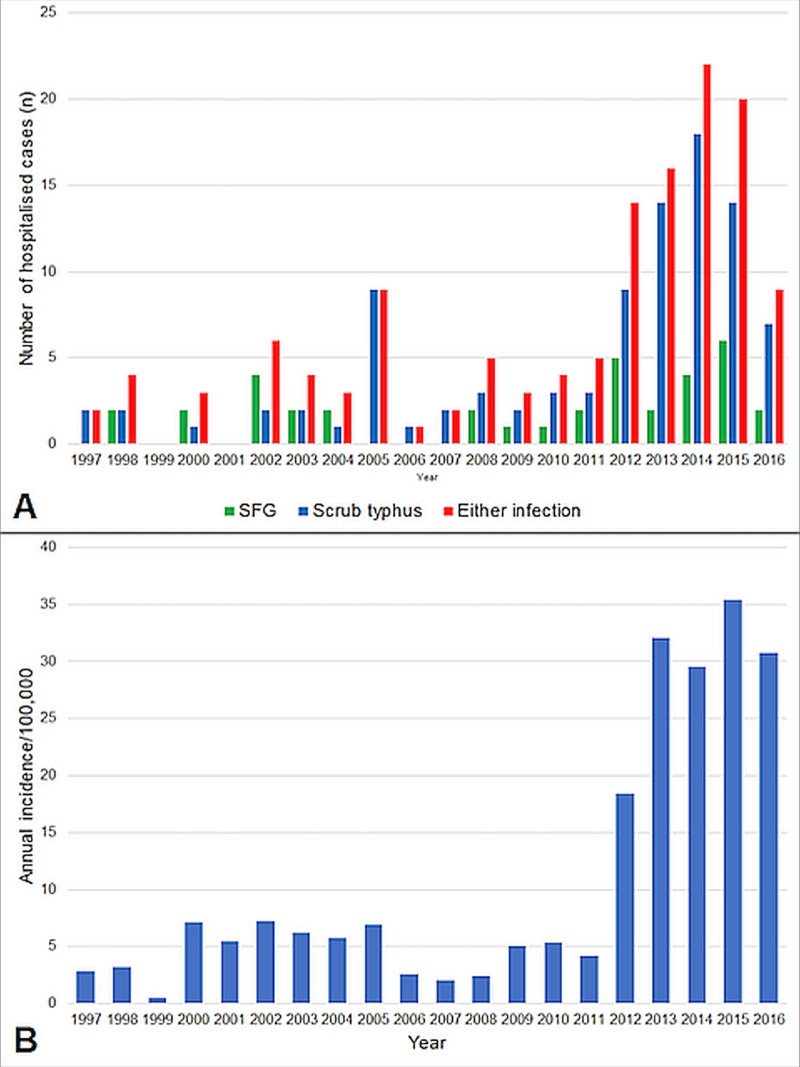
A–The total number of patients with rickettsial infection hospitalized at Cairns Hospital during the study period. B–The annual incidence of rickettsial infections in the Far North Queensland population. Determined using all requests for rickettsial serology from the public health system (positive cases defined using a titre of ≥ 256).

The number of serology requests in the Far North Queensland region increased during the study period (333 in 1998 to 523 in 2016, p for trend = 0.01), but the proportion of tests that were positive also increased (7/333 (2.1%) in 1998 compared with 86/523 (16.4%) in 2016, p for trend = 0.02). To address the possibility of improved diagnostic sensitivity of the serological testing, the last 7 years of the study period–when the same diagnostic test (BioCell Diagnostics) was used–was examined. The proportion of positive tests increased during this period from 13/505 (2.6%) in 2009 to 86/523 (16.4%) in 2016, p for trend = 0.04. The annual incidence of rickettsial infections–positive cases defined as a serological titre ≥ 256 –increased in the region from 3.2/100,000 in 1998 to 30.8/100,000 in 2016 (p = 0.03) (Fig 2).

### Spatial distribution

**Fig 3:**
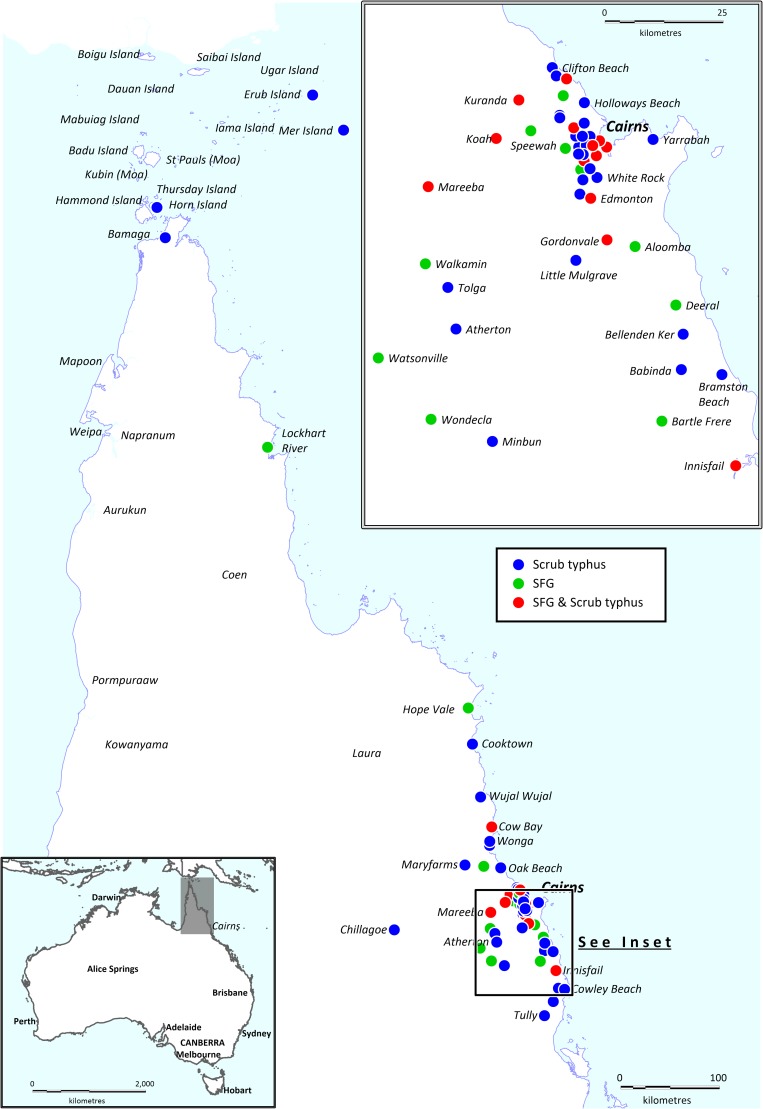
Spatial distribution of cases of rickettsial infections hospitalised at Cairns Hospital during the study period. Note the location of each case is based on the residential address of the patient, not necessarily the location of acquisition, which was frequently impossible to determine in this retrospective study. The map was created using constructed using mapping software (MapInfo version 15.02, Connecticut, USA) using data provided by the State of Queensland (QSpatial). Queensland Place Names—State of Queensland (Department of Natural Resources, Mines and Energy) 2019, available under Creative Commons Attribution 4.0 International licence https://creativecommons.org/licenses/by/4.0/. ‘Coastline and state border–Queensland - State of Queensland (Department of Natural Resources, Mines and Energy) 2019, available under Creative Commons Attribution 4.0 International licence https://creativecommons.org/licenses/by/4.0/. Note Erub Island is also known as Darnley Island.

Cases were widely dispersed across the region (Fig 3). SFG cases occurred as far north as Lockhart River on the Cape York Peninsula. Scrub typhus cases extended further north to the Torres Strait islands.

### Patient demographics

The patients’ median (interquartile range (IQR)) age was 36 (24–52) years; 76 (56%) were male, 15 (13%) were children (age < 16). Only 24 (18%) patients in the cohort had a significant comorbidity. An occupational or recreational risk for exposure was documented in 58 (43%) patients.

### Symptoms and signs

**Fig 4:**
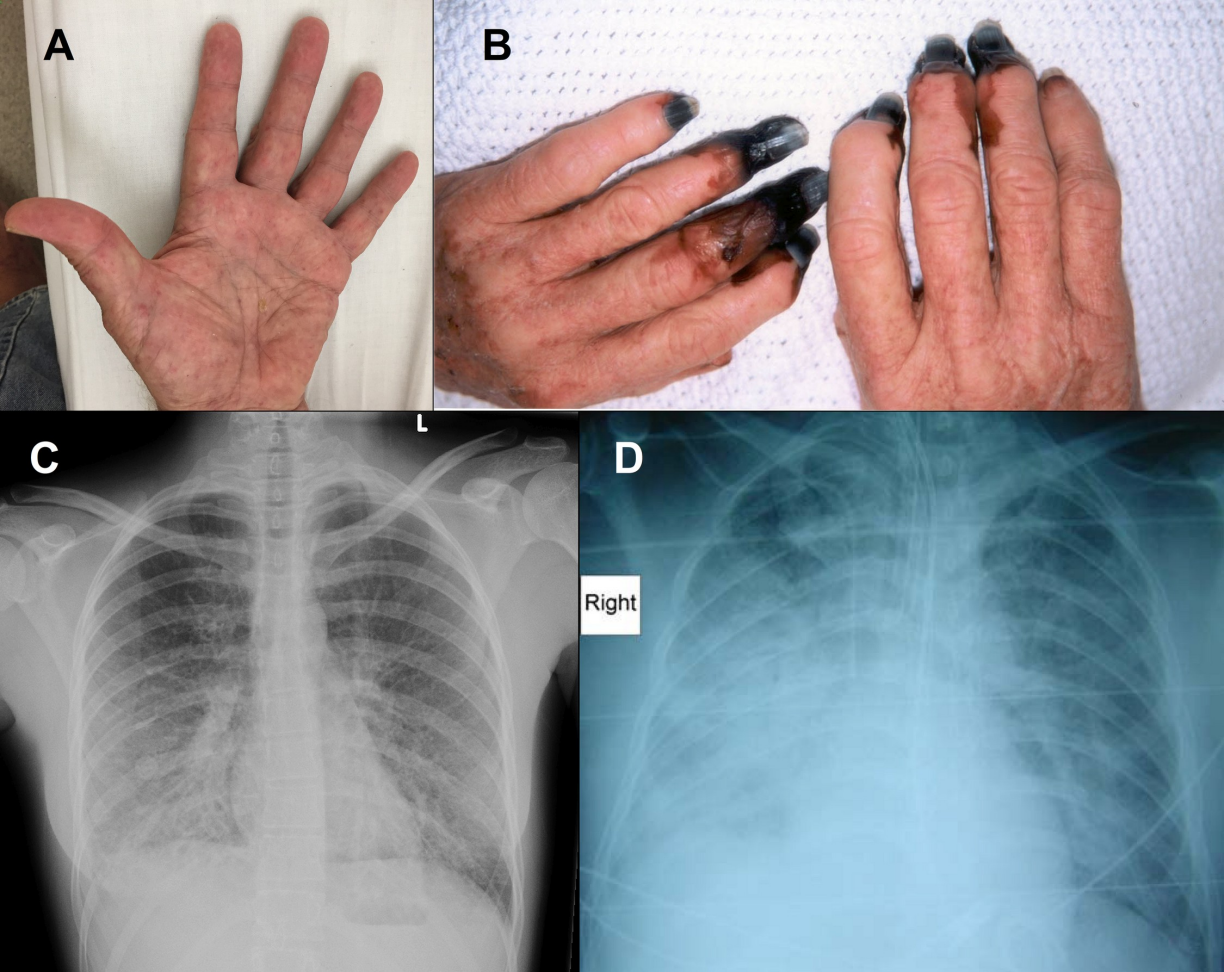
A–Macular petechial rash of the palms in a patient with SFG infection, B–Digital ischemia in a patient with SFG infection; this patient later required digital amputation, C–CXR demonstrating bilateral patchy non-confluent alveolar opacification in a patient with scrub typhus, D–CXR demonstrating right middle and lower lobe pneumonia and early left lower lobe consolidation in a patient with severe SFG infection in ICU. (Panels B and D have been published previously *(10)*).

Fever was present in 130/135 (96%) (Table 1). Headache was more common in those with scrub typhus than those with SFG infection (69/95 (73%) versus 18/37 (49%), p = 0.009). Rash occurred in 54/135 (40%) and was more common in patients with SFG infection (22/37 (59%) versus 31/95 (33%), p = 0.005) (Fig 4). An eschar was identified in 21/135 (15%) and was more common in patients with scrub typhus (19/95 (20%) versus 2/37 (5%), p = 0.04).

**Table 1:** Clinical findings in patients with rickettsial infections at presentation.

	All ^a^ n = 135	Scrub typhus n = 95	SFG n = 37	p ^b^
Male gender	76 (56%)	58 (61%)	16 (43%)	0.06
Age (years)	36 (24–52)	35 (22–46)	45 (28–59)	0.02
Requiring inter-hospital transfer	43 (32%)	31 (33%)	12 (32%)	0.98
Time from symptom onset (days)	6 (2–8)	6 (2–8)	6 (3–8)	0.96
Comorbidity ^c^	24 (18%)	14 (15%)	10 (27%)	0.10
Fever	130 (96%)	91 (96%)	36 (97%)	0.68
Rigors	31 (23%)	23 (24%)	7 (19%)	0.51
Myalgia	103 (76%)	74 (78%)	27 (73%)	0.55
Headache	89 (66%)	69 (73%)	18 (49%)	0.009
Lethargy	63 (47%)	45 (47%)	17 (46%)	0.88
Rash	54 (40%)	31 (33%)	22 (59%)	0.005
Eschar	21 (16%)	19 (20%)	2 (5%)	0.04
Lymphadenopathy	17 (13%)	15 (16%)	2 (5%)	0.11
Hypotension (MAP < 65mmHg)	10 (8%)	9 (10%)	1 (3%)	0.19
Tachycardia (HR ≥ 100 beats/minute)	63 (47%)	44 (47%)	18 (50%)	0.74
Tachypnoea (RR ≥ 22 breaths/minute)	43 (32%)	28 (30%)	14 (39%)	0.32
Impaired consciousness (GCS < 15)	8 (6%)	6 (6%)	2 (5%)	0.84
Hypoxia (pulse oximetry < 95% on RA)	30 (23%)	19 (20%)	11 (31%)	0.19
Any abnormal vital sign	88 (68%)	59 (66%)	28 (78%)	0.18

All numbers represent the absolute number (%) or the median (interquartile range).

^a^ Three cases were undifferentiated rickettsial infections.

^b^ Determined using chi-squared or Kruskal-Wallis test where appropriate.

^c^ Defined as chronic cardiovascular disease (receiving any ongoing treatment for a cardiovascular condition, chronic lung disease (receiving any ongoing treatment for a chronic lung condition), chronic renal disease (a serum creatinine ≥ 150 μmol/L documented before the presentation), immunosuppression (the use of immunosuppressive agents, including corticosteroids, chemotherapy, or immunomodulatory therapies), an active malignancy or the diagnosis of diabetes mellitus.

SFG: Spotted fever group; MAP: mean arterial pressure; HR: heart rate; RR respiratory rate, GCS: Glasgow Coma Scale; RA: room air.

### Laboratory, radiological and echocardiographic findings

Thrombocytopenia, abnormal liver function tests and impaired renal function were common findings (Table 2). A chest x-ray (CXR) was performed in 91/135 (67%); in those who had a CXR performed, abnormal findings were present in 22/63 (35%) with scrub typhus, and 14/28 (50%) with SFG infection (Fig 4). Eighteen (13%) patients had a transthoracic echocardiogram. Small pericardial effusions were noted in 3 (2 scrub typhus cases, 1 SFG case); no other echocardiographic abnormalities were detected.

**Table 2:** The patients’ laboratory findings at presentation.

	All ^a^ n = 135	Scrub typhus n = 95	SFG n = 37	p ^b^
Haemoglobin (g/dL)	135 (122–148)	136 (123–148)	128 (120–149)	0.48
Leucocytes (x 10^9^/L)	8.0 (5.9–11.4)	8.0 (5.9–11.0)	8.0 (5.6–13.4)	0.85
Platelets (x 10^9^/L)	153 (106–206)	155 (112–217)	130 (62–192)	0.02
Platelets <100 x 10^9^/L	30 (22%)	16 (17%)	14 (39%)	0.01
Bilirubin (μmol/L)	17 (12–24)	15 (10–21)	24 (15–32)	0.0006
Bilirubin > 50μmol/L	9 (7%)	5 (5%)	4 (11%)	0.26
ALP (IU/mL)	86 (25–75)	86 (64–133)	129 (69–265)	0.03
GGT (IU/mL)	38 (21–118)	33 (20–85)	92 (25–184)	0.02
ALT (IU/mL)	51 (24–117)	41 (23–96)	86 (36–131)	0.02
AST (IU/mL)	69 (26–156)	55 (25–121)	125 (40–178)	0.04
AST > 100 (IU/mL)	43 (35%)	25 (28%)	18 (51%)	0.02
INR	1.3 (1.1–1.4)	1.3 (1.2–1.4)	1.2 (1.1–1.3)	0.32
APTT (seconds)	34 (31–42)	34 (31–41)	35 (31–46)	0.65
Creatinine (μmol/L)	82 (67–108)	81 (66–105)	97 (70–131)	0.05
Creatinine >120 μmol/L	25 (19%)	15 (16%)	10 (27%)	0.15
Urea (mmol/L)	5.0 (3.7–7.7)	4.6 (3.6–7.4)	6.0 (4.2–11.4)	0.03
C- reactive protein (ng/mL)	83 (30–210)	81 (23–194)	167 (48–329)	0.04
Abnormal chest x-ray	37 (40%)	22 (35%)	14 (50%)	0.18

All numbers represent median (interquartile range).

^a^ Three cases were undifferentiated rickettsial infections.

^b^ Determined using chi-squared or Kruskal-Wallis test where appropriate.

SFG: Spotted fever group; ALP: alkaline phosphatase; GGT: gamma glutamyl transferase ALT: alanine aminotransferase; AST: aspartate aminotransferase; INR: international normalized ratio; APTT: active partial thromboplastin time.

### Management and course of illness

Rickettsial disease was included in admitting clinicians’ initial differential diagnosis in 97/135 (72%); 76 (56%) patients received an antibiotic with anti-rickettsial activity at presentation, while 102 (76%) received an anti-rickettsial antibiotic at some point during their hospitalization. Of the 102 patients who received anti-rickettsial therapy, 99 (97%) adhered to national guidelines [22] for duration and 94 (92%) received therapy within 48 hours of their admission. Doxycycline was used in 93/102 (91%) and azithromycin was used in 11/102 (16%). The median duration of hospitalization was 3 days (IQR 1–6 days, range 0–95 days).

### Severe disease

Eighteen (13%) of the 135 cases required ICU admission, all were adults. A greater proportion of patients with SFG infection required ICU support (8/37 (22%) compared with 10/95 (11%) scrub typhus cases), although this difference did not reach statistical significance (p = 0.10). Patients requiring ICU care were older, had more profound thrombocytopenia, greater liver function test derangement, greater renal impairment, a higher C-reactive protein (CRP) and more likely to have an abnormal CXR (Table 3). Patients requiring ICU care had experienced symptoms for a median (IQR) of 7 (6–10) days prior to presentation, whereas those patients not requiring ICU admission had experienced symptoms for 5 (2–8) days (p = 0.06). Every patient admitted to ICU received anti-rickettsial antibiotic therapy, in 14 (78%) it was within the first 24 hours.

**Table 3:** Characteristics and laboratory/radiology findings in ICU compared to non-ICU patients with a rickettsial infection.

	Number with data	All patients n = 135	ICU admission n = 18	No ICU admission n = 117	p ^a^
Male (%)	135	76 (56%)	9 (50%)	67 (57%)	0.56
Age (years)	135	36 (24–52)	55 (37–67)	35 (23–47)	<0.001
Comorbidity ^b^	135	24 (18%)	6 (33%)	18 (15%)	0.06
Inter-hospital transfer	135	43 (32%)	13 (72%)	30 (26%)	<0.001
Days of symptoms prior to presentation	133	6 (2–8)	7 (6–10)	5 (2–8)	0.06
Platelets (x10^9^/L)	134	153 (106–206)	115 (76–155)	156 (108–217)	0.02
Bilirubin (μmol/L)	130	17 (12–24)	25 (16–38)	16 (11–23)	0.007
ALP (IU/mL)	131	86 (66–187)	195 (86–265)	86 (65–151)	0.006
GGT (IU/mL)	131	38 (21–118)	122 (54–213)	33 (20–92)	<0.001
ALT (IU/mL)	131	51 (24–117)	119 (75–197)	41 (23–108)	<0.001
AST (IU/mL)	125	69 (26–156)	185 (110–334)	54 (24–123)	<0.001
International normalized ratio	40	1.3 (1.1–1.4)	1.4 (1.2–1.6)	1.2 (1.1–1.3)	0.05
APTT (seconds)	38	34 (31–42)	38 (32–44)	33 (31–38)	0.21
Creatinine (μmol/L)	134	82 (67–108)	130 (94–212)	81 (66–100)	<0.001
Urea (mmol/L)	134	5.0 (3.7–7.7)	10.5 (6.2–19.2)	4.5 (3.5–6.8)	<0.001
Abnormal chest x-ray	134	37 (40%)	12 (71%)	25 (33%)	0.004
C-reactive protein (ng/mL)	79	83 (30–210)	270 (188–347)	74 (27–188)	0.001
Appropriate antibiotics on admission	135	76 (56%)	14 (78%)	62 (53%)	0.048
Presence of an eschar	135	21 (16%)	16 (14%)	5 (28%)	0.12
Infectious diseases physician consultation	135	42 (31%)	12 (67%)	30 (21%)	<0.001

^a^ Determined using Fisher’s exact or Kruskal-Wallis test where appropriate.

^b^ Comorbidity: chronic cardiovascular disease (receiving any ongoing treatment for a cardiovascular condition, chronic lung disease (receiving any ongoing treatment for a chronic lung condition), chronic renal disease (a serum creatinine ≥ 150 μmol/L documented before the presentation), immunosuppression (the use of immunosuppressive agents, including corticosteroids, chemotherapy, or immunomodulatory therapies), an active malignancy or the diagnosis of diabetes mellitus.

ALP: alkaline phosphatase; GGT: gamma glutamyl transferase ALT: alanine aminotransferase; AST: aspartate aminotransferase; APTT: active partial thromboplastin time.

### Mortality and morbidity

There was one death, in a patient with SFG infection. A 55-year-old farmer, without significant co-morbidities, presented with a 6-day history of fever, headache and myalgia, after a possible tick bite two weeks earlier. He was hemodynamically unstable on presentation with acute kidney injury, elevated transaminases and laboratory evidence of disseminated intravascular coagulation (DIC). He was transferred to ICU where despite vasopressor support, renal replacement therapy (RRT), mechanical ventilation and antibiotic therapy (initially with piperacillin-tazobactam and doxycycline, subsequently escalated to meropenem, vancomycin and azithromycin) he progressed to multi-organ failure and died less than 48 hours after presentation.

Two patients with SFG infection had disabling sequelae, developing digital ischemia requiring amputation (Fig 4). Both patients had evidence of purpura fulminans (skin necrosis and DIC) and both required RRT and mechanical ventilation for survival. Overall 3/37 (8%) patients with SFG infection died or had permanent disability compared with 0/95 patients with scrub typhus (p = 0.02) (Table 4).

**Table 4:** Patients with a complicated clinical course.

	Scrub typhus n = 95 (%)	SFG infection n = 37 (%)	p ^a^
ICU Admission	10 (11%)	8 (22%)	0.16
Died	0	1 (3%)	0.28
Permanent disability	0	2 (5%)	0.08
Death/permanent disability	0	3 (8%)	0.02
Mechanical ventilation	7 (7%)	4 (11%)	0.50
RRT	2 (2%)	3 (8%)	0.13
Vasopressor therapy	8 (8%)	7 (19%)	0.12
Supportive care ^b^	9 (9%)	7 (19%)	0.15

All numbers represent absolute number (percentage).

^a^ Determined using Fisher’s exact test

ICU: Intensive Care Unit; RRT: Renal replacement therapy

^b^ Required mechanical ventilation, RRT or vasopressor support.

SFG: Spotted fever group; ICU: intensive care unit; RRT: renal replacement therapy.

### Prediction of severe disease

None of the 15 children required ICU admission. Among the 120 adults, there were 5 variables which, when determined on admission, had an AUROC > 0.7 in univariate analysis for predicting subsequent ICU admission: respiratory rate (AUROC 0.87, 95% CI:0.79–0.94), CRP (AUROC 0.82, 95% CI:0.68–0.95), plasma aspartate aminotransferase (AUROC 0.82, 95% CI:0.73–0.92), plasma creatinine (AUROC 0.75, 95% CI: 0.62–0.88) and age (AUROC 0.72, 95% CI: 0.59–0.86). Binary variables were created for these 5 continuous variables using reference ranges and common clinical usage (Table 5). In multivariate analysis, 2 of these variables–a respiratory rate ≥ 22 (odds ratio (OR): 13.2 (3.8–46.0), p < 0.001, and a plasma creatinine > 120 μmol/L (OR (95% CI): 3.5 (95% CI 1.03–12.0), p = 0.04)–were found to be independently predictive. If only the clinical variables of age and respiratory rate in adult patients were examined in multivariate analysis–the odds ratio of a respiratory rate ≥ 22 had an odds ratio (OR) for ICU admission of 11.6 (95% CI: 3.3–40.5, p < 0.001) while an age ≥50 had an OR for ICU admission of 5.1 (95% CI:1.6–16.2, p = 0.006). If an adult patient was <50 years and had a respiratory rate of < 22 on presentation to hospital, there was a negative predictive value (NPV) for ICU admission of 97% (95% CI 89–100). Meanwhile, if an adult ≥ 50 had a respiratory rate ≥ 22 on presentation to hospital, the positive predictive value (PPV) for ICU admission was 62% (95% CI 32–86).

**Table 5:** Ability of variables to predict adult patients’ subsequent requirement for Intensive Care Unit admission.

Variable ^a^	Number with data	Odds ratio	95% CI	p value
Age ≥ 50 years	120	3.5	(1.2–9.7)	0.02
Respiratory rate ≥ 22 breaths/minute	118	14.9	(4.4–50.5)	<0.001
Plasma creatinine > 120μmol/L	119	5.3	(1.8–15.4)	0.002
C-reactive protein ≥ 100 (ng/mL)	69	8.6	(1.0–72.7)	0.049
AST ≥ 100 (IU/mL)	112	8.2	(2.5–26.3)	0.001

^a^ Variables selected based on an area under the receiving operator characteristic curve of >0.7. Cut-offs based on common clinical usage. Univariate analysis is presented.

No children (age <16 years) required ICU admission.

AST: aspartate aminotransferase

A qSOFA score could be calculated in 117 adults: a high qSOFA score (≥ 2) was present in 12 (10%) and had an OR of 19 (95% CI: 4.8–74.5) for ICU admission (p < 0.001). A NEWS2 score could be calculated in 119 adults, a high NEWS score (≥7) was present in 21 (18%) and had an OR of 14.3 (95% CI:4.5–45.2) for ICU admission (p < 0.001). The NPV of a low qSOFA score (<2) and a low NEWS2 score (<7) for ICU admission were 91% (95% CI: 83–95) and 93% (95% CI: 86–97) respectively. The ability of a high qSOFA or high NEWS2 score to predict death/disability and specific organ dysfunction are presented in Table 6.

**Table 6:** Ability of the qSOFA and NEWS2 scores on admission to predict subsequent death or specific organ dysfunction in adults.

Variable	Number	High qSOFA score Odds ratio (95% CI)	p	High NEWS2 score Odds ratio (95% CI)	p
Death or disability	3	20.8 (1.7–250.0)	0.02	10.2 (0.9–118.4)	0.06
Renal replacement therapy	5	6.8 (1.01–45.6)	0.048	8 (1.2–51.3)	0.03
Mechanical ventilation	11	35.4 (7.7–161.9)	<0.001	36 (6.9–186.6)	<0.001
Vasopressor requirement	15	10.7 (2.8–40.0)	<0.001	11.5 (3.5–38.0)	<0.001

## Discussion

This series demonstrates the significant–and increasing–clinical burden of rickettsial disease in tropical Queensland, Australia. While scrub typhus is seen more commonly in the region, SFG infections appear to have a more severe clinical phenotype. In this series, 22% of SFG cases required ICU admission, suggesting that severe disease may be more common in patients with SFG infection than previously believed [9]. Over 10% of scrub typhus cases also required ICU support. However, despite the frequency of severe disease, there was only a single death in the series. This is likely due to the high local awareness of rickettsial infections, resulting in early initiation of appropriate antibiotic therapy and prompt access to sophisticated supportive care in the well-resourced Australian health system.

The local incidence of rickettsial infections increased almost tenfold over the study period. While improved diagnostic techniques have been hypothesized to account for increased incidence in some countries [28], this is unlikely to be the case in this series; the same serological techniques have been used since 2009 with the most striking increase in cases occurring from 2012 onwards. Indeed, this series is likely to significantly underestimate the clinical burden of rickettsial disease given the challenges in establishing the diagnosis [28] and the high frequency with which local patients are treated empirically without diagnostic testing [9].

The population grew by 65,725 (31%) during the study period [21], leading to expansion of residential areas into the urban-rural fringe, with the potential for increased interaction with an environment that is an ideal habitat for mites and ticks [29–31]. The region contains over 70 national parks and has an active agricultural sector. Indeed, 43% of the cases had a recorded occupational or recreational risk for mite or tick exposure, which given the retrospective nature of the study, is almost certainly an underestimate.

Changes in vector distribution may also have contributed to the increased incidence and there were cases in the northern part of the region where SFG infection has not previously been identified [31]. Humans entering “mite islands”–where there are many vertebrate reservoirs in an optimal ecosystem for mites–have an increased risk for scrub typhus [19, 32]. An increase in the number and size of these islands may increase the risk of human disease. It is uncertain what impact climate change would have over this relatively short time period, although it is intuitive that the range and distribution of vertebrate hosts carrying infected mites and ticks may change with the predicted future alterations in rainfall and temperature [11, 33].

Scrub typhus was more common than SFG infections during the study period, but SFG infections had a more severe clinical phenotype, echoing the findings of another series from southern Queensland where 11% had severe disease [34]. Our cohort contains the third-ever reported fatality from SFG infection in Australia [9, 35], while 22% of the SFG cases required ICU care. Renal disease was also relatively common in patients with SFG infection in this series. New renal impairment developed in 24% of the cohort and 8% required RRT. These observations suggest that significant renal impairment–and severe disease–may be more common in SFG infections than previously believed [9, 34].

Although more than 10% of the scrub typhus cases in the cohort also required ICU care, the disease caused no deaths, which contrasts with the high mortality rates reported from some international studies [6, 8]. Compared with those series, the patients in this cohort were generally young with few comorbidities and presented relatively early where they usually received appropriate treatment promptly [6, 8]. Although rickettsial infections–particularly scrub typhus–are described frequently as neglected diseases [33, 36], the diagnosis was included in the initial differential in 72% of cases and most patients received appropriate therapy within 24 hours of their presentation. Access to sophisticated ICU support–unavailable in most areas of the world where rickettsial infections are endemic–also no doubt contributed to the excellent outcomes. However, the fact that two-thirds of the patients admitted to ICU had no significant comorbidities, emphasizes that these infections can cause critical illness in otherwise well individuals.

While there have been reports of doxycycline resistance in scrub typhus in some locations [19, 37] and the suggestion that azithromycin is superior therapy [38], doxycycline appeared to have good efficacy in this series. The single death was most likely the result of a delayed presentation rather than doxycycline resistance.

The clinical manifestations of rickettsial diseases can vary from a mild self-limiting illness to life-threatening multi-organ failure [8]. This has led to clinicians calling for the development of a tool that might expedite the identification of patients at risk of disease progression [11]. The fact that simple, clinical assessment on admission had an NPV for subsequent ICU admission of greater than 95% in the cohort is noteworthy and is particularly attractive for clinicians in rural and remote settings, where the majority of rickettsial infections occur. This observation echoes the findings of other studies that have proven the utility of simple clinical assessment in predicting the progression of infectious diseases in resource-limited settings [16, 39–42]. Scoring systems with a similar emphasis on “the vital signs”–such as the NEWS2 score–can facilitate the identification of the high-risk patient, by even inexperienced health workers, whatever the underlying illness [24, 43]. A similar tool has been used locally since 2012 to assess the deteriorating patient [44], standardizing the approach to the sick patient, whether they are presenting with rickettsial disease, another infection or a non-communicable illness.

In this study, clinical scores that can be calculated on presentation, had an excellent NPV for subsequent severe disease. Even in Australia’s well-resourced health system, many of the cases of rickettsial disease are seen in rural and remote settings where clinicians will frequently have to wait greater than 24 hours to access laboratory results that have prognostic utility. These clinical prediction tools therefore provide reassurance that the patient is less likely to develop severe disease and can continue to be managed safely in the peripheral facility, or even discharged home. The scores, which can be determined in minutes at the bedside, may also have utility in patient triage in low-resource settings when rickettsial diseases are also endemic and where laboratory support is frequently absent.

This study highlights the difficulties in diagnosing rickettsial infections [28]. Serology was the mainstay of diagnosis, with most cases diagnosed with a single positive titre. As antibodies may not be detected in the acute phase of rickettsial illness, a single test will underestimate the burden of disease. Additionally, there is no consensus on the optimal cut-off titre for diagnosis [28, 45]. There is serological cross-reactivity between different SFG-rickettsioses and although *R*. *australis* infection is considered more common [9, 29, 30], *Rickettsia honei* strain “*marmionii*” has been reported in the region [31]; it was therefore not possible to distinguish between these two pathogens in this study. Serological cross-reaction between the SFG and the typhus group rickettsiae also means that some of the cases in this series may have represented murine typhus. However, none of the tests that were performed for the typhus group in the region during the study period had a titre >1:64, and the disease, transmitted by fleas, is seen extremely rarely in 21^st^ Century Australia [9, 46]. Interpretation of serology is especially challenging in endemic areas where low titres can represent previous exposure or subclinical infection [45, 47]. Meanwhile, other infectious diseases and autoimmune conditions can also generate false positive serological results [29, 30, 36, 48]. Rickettsial PCR on blood can facilitate diagnosis, especially early in the disease course when serology may be negative, while PCR of eschars/skin lesions has a high sensitivity [28]. Unfortunately, the expense of PCR testing precludes its use in resource-poor settings and even in Australia, the processing time limits its clinical utility. Serology testing can also have a significant turnaround time, with tests often batched in the Australian health system to save costs. Indeed, delays in the availability of laboratory results explains why at least some of the cohort did not receive anti-rickettsial antimicrobial therapy, despite serology that suggested the diagnosis. An inexpensive and reliable point-of-care test would address many of these issues, although the sensitivity of presently available tests is disappointing [5].

The study has several limitations. Its retrospective nature means that the data are incomplete, with clinical manifestations under-reported or incompletely described. The description of the geographical distribution of cases is also imperfect as the map (Fig 3) presents the residential address of the patients, rather than the location of likely exposure, which was rarely available in the medical record. Clinical cases included in this series were managed in a tertiary-referral hospital, which serves to both underestimate the diseases’ incidence and overestimate their severity. A minority of the cohort had the diagnosis confirmed by PCR or a fourfold rise in serology; most were diagnosed with a single serological titre, although patients were only included in the study if they satisfied pre-specified, conservative criteria, similar to those used in the international literature [34, 49, 50]. Scrub typhus and SFG infections are presented together and some rickettsiologists may take issue with this approach. However, the infections have similar epidemiology, pathogenesis, presentation and the same treatment and hence, from a pragmatic, clinical perspective we felt that it was reasonable to consider them together.

### Conclusions

This report highlights an increasing incidence of scrub typhus and SFG infections in tropical Australia. It also suggests that clinical manifestations of SFG infections may be more severe than previously believed. Although they are neglected diseases globally and in absolute terms, an uncommon cause of hospitalisation in tropical Australia, local clinicians appear to have a good awareness of the infections, which have an excellent prognosis when treated promptly in a well-resourced heath setting. Simple, bedside clinical assessment appears helpful in identifying the patient at high risk of subsequent deterioration and may be useful for clinicians managing these patients in resource-limited settings.

## Supporting information

S1 Checklist STROBE checklist. (DOC)

S1 Table Summary of the methods for rickettsia serological diagnosis between 1997 –present. (DOCX)
