# Supplementary material for: The epidemiology and clinical features of rickettsial diseases in North Queensland, Australia: Implications for patient identification and management
Source: PLoS Negl Trop Dis. 2019 Jul 18;13(7):e0007583. doi: 10.1371/journal.pntd.0007583 (PMC6667154; doi:10.1371/journal.pntd.0007583)
Supplement: S1 Table — (DOCX) [file pntd.0007583.s002.docx]

**Supplementary table 1.** Summary of the methods for rickettsia serological diagnosis between 1997 – present

|  | Method for rickettsia serological diagnosis |
| --- | --- |
| 1997 – mid-1998 | In-house IFA (FSS):   - *R. australis* Ig Total - *O. tsutsugamushi* Gilliam Ig Total - *O. tsutsugamushi* Ig Total |
| Mid-1998 – mid-1999 | In-house IFA (FSS):   - Spotted fever group Ig Total - Typhus group Ig Total - Scrub typhus group Ig Total |
| Mid-1999 – mid-2009 | EIA (PanBio) screening:   - Spotted fever group IgM - Scrub typhus group IgM   For positive results confirmatory IFA (FSS):   - *R. australis* Ig Total - *O. tsutsugamushi* Ig Total |
| Mid-2009 - present | IFA (BioCell):   - *R. rickettsia** Ig Total - *O. tsutsugamushi* Ig Total |

**R. rickettsia* used as the surrogate for Spotted Fever Group, due to cross reactivity with *R. australis*.

The serological cut-off titre for a positive result was changed from > 64 to > 128 in January 2015

IFA: immunofluorescence assay; EIA: enzyme immunoassay.

FSS: Forensic and Scientific Services (Pathology Queensland).
